# A global sea state dataset from spaceborne synthetic aperture radar wave mode data

**DOI:** 10.1038/s41597-020-00601-3

**Published:** 2020-08-07

**Authors:** Xiao-Ming Li, BingQing Huang

**Affiliations:** 1 Aerospace Information Research Institute, Chinese Academy of Sciences, 100094 Beijing, P. R. China; 2 University of Chinese Academy of Sciences, 100049 Beijing, P. R. China

**Keywords:** Physical oceanography, Physical oceanography, Geography, Renewable energy

## Abstract

This dataset consists of integral sea state parameters of significant wave height (SWH) and mean wave period (zero-upcrossing mean wave period, MWP) data derived from the advanced synthetic aperture radar (ASAR) onboard the ENVISAT satellite over its full life cycle (2002–2012) covering the global ocean. Both parameters are calibrated and validated against buoy data. A cross-validation between the ASAR SWH and radar altimeter (RA) data is also performed to ensure that the SAR-derived wave height data are of the same quality as the RA data. These data are stored in the standard NetCDF format, which are produced for each ASAR wave mode Level1B data provided by the European Space Agency. This is the first time that a full sea state product in terms of both the SWH and MWP has been derived from spaceborne SAR data over the global ocean for a decadal temporal scale.

## Abstract (summary)

**Table:** 

Measurement(s)	sea state
Technology Type(s)	satellite imaging of a planet • synthetic aperture radar
Factor Type(s)	year of data collection
Sample Characteristic - Environment	ocean
Sample Characteristic - Location	Earth (planet)

Machine-accessible metadata file describing the reported data: 10.6084/m9.figshare.12611618

## Background & Summary

The sea state is one of the key parameters of the “essential climate variables” (ECVs) defined by the Global Climate Observing System (GCOS) to meet the requirements of the climate change community. Spaceborne radar measurements of the sea state in terms of the significant wave height (SWH) and mean wave period (MWP), particularly from radar altimeters (RAs), have been available for a few decades^1^. Long-term RA measurements can reflect some wave height trends in the global oceans, and these trends might be associated with climate change^2^. Another radar sensor capable of measuring the sea state is known as spaceborne synthetic aperture radar (SAR), which became available at the same time as RAs; consequently, both instruments were on board the Seasat^3^ satellite launched in 1978. However, unlike nadir-looking RAs, SAR is a side-looking radar, which allows SAR to image large surface areas. Additionally, SAR can achieve a high spatial resolution in the azimuth (flight) direction through the “aperture synthetizing” technique^4^. In principle, spaceborne SAR should be able to effectively measure the sea state from space, as this technology images sea surface waves in two dimensions^5^, at a high spatial resolution. However, as surface waves are in motion during the SAR imaging time (i.e., water particles are moving either toward or away from the radar system), the high-frequency components of ocean waves are missed (the “cut-off” effect), and the distortion of the spectrum occurs during the imaging process of SAR^6,7^. Therefore, the SAR imaging of surface gravity waves is generally considered a nonlinear process^8^, complicating the retrieval of ocean wave parameters from SAR data. Two-dimensional wave spectra predicted by ocean wave modeling (e.g., WAM^9^) or derived from other sources^10^ must commonly be used as a priori information (also called the “first guess”) in the retrieval^11^ to compensate for the lost and distorted ocean wave information during SAR imaging. However, as a result of this compensatory approach, the retrieval of ocean wave parameters from SAR data has to rely on the priori information, which significantly limits SAR as an independent remote sensing instrument that can measure the sea state.

The wave mode (WM), which is dedicated to measurements of ocean wave, is a unique imaging mode of SAR. Although the WM covers a relatively small area of the sea surface (approximately 6 km by 10 km), these data are automatically acquired by spaceborne SAR over the global oceans. From the European Remote Sensing Satellite missions (ERS-1, 1991–2000 and ERS-2, 1995–2011)^12–15^ to the Environment Satellite (ENVISAT) mission (2002–2012)^16,17^ and the current Sentinel-1A/1B (2014 -)^18^ and the Chinese Gaofen-3 (2016 -) missions^19^, WM data have been available for nearly 30 years and will continue to be acquired into the future, constituting a valuable dataset for global sea state measurements. On the basis of SAR WM data, some interesting investigations of global ocean waves, particularly with respect to the dynamics of ocean swells^20–22^, have been reported. Such analyses can be performed because ocean swells are generally considered linearly or quasi-linearly imaged by SAR; thus, the abovementioned nonlinear inversion process can be degraded to a quasilinear approach^23,24^, in which case a priori information is no longer needed. However, such a quasilinear inversion cannot yield full sea state parameters of both windsea (wind waves) and swell, and instead yields the sea state parameters of swell, or more accurately called the parameters of the ocean wave components imaged by spaceborne SAR. Therefore, to overcome such a weakness, various parametric models that directly relate SAR-measured sea surface radar backscatter (radar cross section) to the full sea state parameters of SWH and MWP of both windsea and swell have been proposed^14,17,18^, which also do not need a priori information and can provide independent SAR measurements of global ocean waves. Here, we developed a global sea state dataset from the WM data acquired by the advanced synthetic aperture radar (ASAR) onboard the satellite ENVISAT from 2002 to 2012 based on the parametric model “CWAVE_ENV”^17^. This is the first time that a global ocean dataset of full sea state parameters in terms of both SWH and MWP in a decadal temporal scale has become publicly available based on spaceborne SAR data and we believe that this dataset, in conjunction with RA datasets that are widely exploited at present, is valuable for global observations of ocean waves. On the other hand, full sea state parameters also become available in the Sentinel-1 WM data based on the similar retrieval method to the CWAVE type algorithms. Therefore, by combining both the historical ASAR WM ocean wave product and the current continuously obtained Sentinel-1 WM product, one can expect a long-term spaceborne SAR sea state dataset available for global ocean observations.

## Methods

### ASAR WM data

In the ERS-1 and ERS-2 missions, the SAR WM data were publicly available in the formation of two-dimensional image spectra in discrete formats, i.e. allocating the image spectrum energy in numbers of directional and frequency bins^25^. Beginning with the ENVISAT mission, the ASAR WM data in single look complex format^26^ (i.e., consisting of a real part R_*e*_ and an imaginary part I_*m*_) were provided to users; these data record both the magnitude and phase of the returned radar signals. The SAR image intensity (_*I*_) is therefore calculated as $I={R}_{e}^{2}+{I}_{m}^{2}$. By performing a radiometric calibration of intensity data, the normalized radar cross section, denoted *σ*_0_, can be obtained and then used to retrieve sea state parameters.

**Fig. 1:**
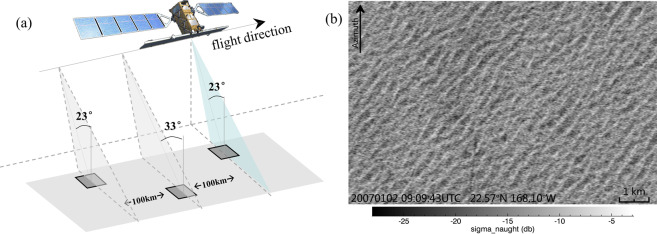
(**a**) Geometry of the ASAR WM data acquisitions. Note that the WM data with an incidence angle of 33° acquired by ASAR were only available during experiments (refer to the main text for details). (**b**) is an example of ASAR WM images acquired over the ocean. The acquisition date and location are marked on the image.

ASAR WM data have a spatial coverage ranging from 6 km × 5 km to 10 km × 5 km over the sea surface. The distance between two consecutive acquisitions of WM data is 100 km, which could be seen from the image geometry of ASAR WM in Fig. 1(a). One example of ASAR WM data acquired over the ocean is shown in Fig. 1(b), which clearly displays patterns of ocean waves (swells).

The parametric model “CWAVE_ENV” was applied to the ASAR WM data to generate the sea state parameters of SWH (*H*_*s*_) and MWP (*T*_*m*02_). This type of parametric model was first proposed for the reprocessed ERS-2 WM data^14^; the name “CWAVE” indicates the use of a C-band (SAR) wave retrieval algorithm, like the widely used C-band geophysical model function “CMOD”^27^ to retrieve sea surface wind fields from scatterometer and SAR data. Because the development and validation of parametric models have been described in detail in previous studies^14,17^, only the rationale for using the parametric model is discussed here.

Although imaging mechanisms of ocean surface gravity waves by SAR remain to be further investigated, the measured radar backscatter from the sea surface is closely related to various sea state parameters (denoted *W*) through relations with a set of parameters (expressed as a vector, ${\boldsymbol{S}}({s}_{1},\ldots ,{s}_{ns})$). These parameters can be directly derived from SAR data, as expressed in Eq. (1).

1 $$W={a}_{0}+\sum _{1\le i\le {n}_{s}}{a}_{i}{s}_{i}+\sum _{1\le i\le j\le {n}_{s}}{a}_{i,j}{s}_{i}{s}_{j}$$

In the above model, the sea state parameter *W* is expressed as a linear combination of a number of *n*_*s*_ ASAR image parameters ${\boldsymbol{S}}({s}_{1},\ldots ,{s}_{ns})$ with the extended coefficient vector ${\boldsymbol{A}}({a}_{0},\ldots ,{a}_{ns},{a}_{11},\ldots ,{a}_{{n}_{s}}{a}_{{n}_{s}})$. To also include nonlinearities as well as possible coupling among different parameters, a quadratic term is added to the equation (the third term in the equation). There are 22 ASAR image parameters (i.e., *n*_*s*_ = 22) used in the CWAVE_ENV model. Two parameters of the normalized radar cross section *σ*_0_ and the variance *cvsr*, are directly calculated from the intensity data. The remaining 20 parameters are derived from the FFT spectrum of the ASAR WM intensity image. The major reason for using the spectral parameters is that the traditional nonlinear or quasi-linear retrievals connect the SAR image spectrum with two-dimensional ocean wave (or swell) spectra. On the other hand, *σ*_0_ is closely related with the wind speed (e.g., represented by the CMOD functions), and therefore, the information of windsea on SAR images is also involved in this equation. This is the general rationale that the function can represent both swell and windsea information.

The least-square minimization approach is used to determine the coefficient vector *A* which consists of a number of *n*_*A*_ coefficients, as defined in Eq. (2), where $\left({w}^{(1)},{{\boldsymbol{S}}}^{(1)}\right),\ldots ,\left({w}^{(N)},\ldots ,{{\boldsymbol{S}}}^{(N)}\right)$ represents the available data pairs of SAR image parameters and the collocated tuning dataset of the integral sea state parameter (e.g., SWH or MWP).

2 $${J}_{cost}(A)=\mathop{\sum }\limits_{j=1}^{N}{\left({w}^{(j)}-\mathop{\sum }\limits_{i=0}^{{n}_{A}-1}{{\boldsymbol{A}}}_{i}{{\boldsymbol{S}}}_{i}^{j}\right)}^{2}$$

After the coefficient vector *A* is determined, one can derive the SWH or MWP directly from ASAR WM data using Eq. (1). The preliminary validation of the ASAR-derived *h*_*s*_ values using the CWAVE_ENV algorithm was conducted for a two-month (January and February 2017) dataset. Comparisons with the National Data Buoy Center (NDBC) *in situ* buoy measurements yielded a bias of 0.06 m and a root-mean-squared-error (RMSE) of 0.70 m^17^. Here, we applied this parametric model to the entire dataset of the ASAR WM data of its full life cycle.

The entire ENVISAT mission ranged from March 2002 to April 2012. The ASAR data that we received from the European Space Agency (ESA) cover the period from December 2002 to April 2012. During the lifetime of ENVISAT, the ASAR instrument acquired WM data in vertical-vertical (VV) polarization with an incidence angle of 23°, except during two experimental periods, in which the acquired WM data had an incidence angle of approximately 33°. The first period ranged from January 24^th^ to February 6^th^, 2007, and the second one ranged from March 6^th^ to March 13^th^, 2007. From January 24^th^ to January 30^th^, 2007, the WM data were acquired in horizontal-horizontal (HH) polarization. In addition to excluding the WM data acquired during these two experimental periods, the following criteria were applied to further screen the data.

(i) The ASAR WM data acquired in polar regions were excluded from further processing because they might be affected by sea ice; thus, only the data acquired between 65°S and 70°N were used to generate sea state parameters.
(ii) Although ASAR WM images have a relatively small spatial coverage compared with images acquired in other modes, e.g., the imaging mode and wide swath mode, the WM images are also affected by other sea surface features not related with ocean waves, e.g., oil spills, atmospheric features, and bright targets. To select only ASAR WM images that display a homogeneous sea surface (e.g., the case shown in Fig. 1b) and derive sea state parameters, some parameters were used for automatic detection. We previously used the “homogeneity factor”^28^ to classify ASAR WM images into homogeneous and inhomogeneous classes; if the sea surface is purely homogeneous, this factor is equal to 1. Through the visual inspection of large amounts of both ERS-2/SAR and ENVISAT/ASAR WM data, the homogeneity factor was set to 1.05 as a threshold for selecting appropriate ASAR WM data for retrieval. Approximately 94.42% of the data have a homogeneity factor lower than 1.05, which are treated as good data for deriving sea state parameters. However, the trade-off is that for WM data with a homogeneity parameter higher than 1.05 may also present an acceptable situation of retrieval. Therefore, we lowered the threshold of the homogeneity factor to 1.50 in order to process more data to sea state parameters, but the data with a homogeneity factor in the range between1.05 and 1.50 are flagged as “suspect” for further investigation, which is described in detail in Data Records. It should be noted that only the WM data with a homogeneity parameter less than 1.05 were used for calibration and validation presented in the following. After the aforementioned preprocessing steps, approximately 6.69 million ASAR WM data were used to generate global ocean wave parameters.

### *In situ* buoy data

*In situ* buoy measurements of sea state parameters were used to validate and calibrate the retrieved SWH and MWP based on the ASAR WM data. The GlobWave project (http://globwave.ifremer.fr/) collected a large amount of *in situ* buoy data from several buoy networks. Among the different buoy datasets, it is found that the one provided by the European Center for Medium-Range Weather Forecasts (ECMWF) contains more data (649 buoys collected between 2002 and 2012) than any of the other datasets. It is a comprehensive collection of buoy data from various networks including NDBC, the Marine Environmental Data Section (MEDS), the Coastal Data Information Program (CDIP) and others. Therefore, we selected the ECMWF-provided buoy data (hereafter referred to as “ECMWF buoy data”) for the evaluation and calibration of the ASAR-derived SWH.

The ASAR-retrieved MWP is the zero-upcrossing mean wave period (*T*_*m*02_, also often denoted *T*_*z*_), as defined in Eq. 3. One can find that both the definitions of *H*_*s*_ and *T*_*m*02_ are in a consistent manner relating to zero-upcrossing waves. Both are the two widely used parameters to describe sea state. We found that many recorded MWP data in the ECMWF buoy dataset are with values of zero, in contrast, the corresponding NDBC spectral data are normal. Therefore, we used the NDBC two-dimensional buoy spectrum (also accessed from the GlobWave data portal, hereafter called “NDBC buoy data”) to calculate *T*_*m*02_ for comparison with the ASAR-retrieved MWP. The quality flag of the NDBC buoy spectral data in the GlobWave data portal is named *spectral_wave_density_qc_level*. The values of this flag are 0, 1, 2, 3 and 4, which represent *unknown*, *unprocessed*, *bad*, *suspect* and *good*, respectively. We used only the good wave spectral data for calibration and validation.

3 $${T}_{m02}=\sqrt{{m}_{0}/{m}_{2}}$$

4 $${m}_{n}=\sum _{i}\,{f}_{i}^{n}{S}_{i}\Delta {f}_{i}$$

In the above equations, m_*n*_ is the *n*^*th*^ spectral moment, *f*_*i*_ is the *i*^*th*^ discrete frequency, *Δf*_*i*_ is the width of the *i*^*th*^ discrete frequency and *S*_*i*_ is the spectral density over the *i*^*th*^ frequency.

**Fig. 2:**
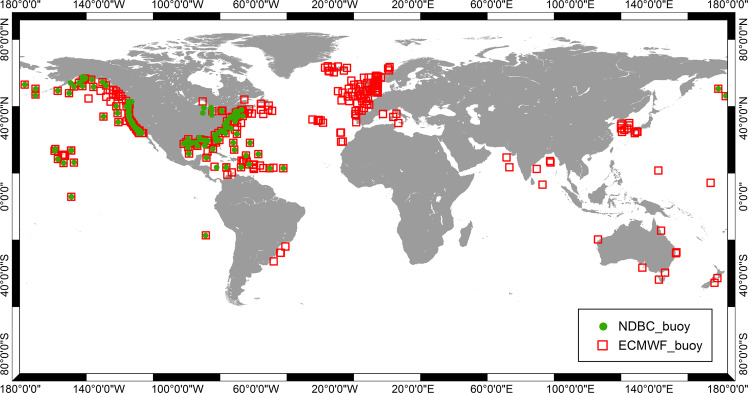
Locations of the collocated ECMWF buoys (red squares) and NDBC buoys (green dots).

Both the ECMWF and NDBC buoy data were collocated with the ASAR WM data following the criteria that the temporal difference is less than 30 minutes and the spatial distance is less than 100 km. For cases in which several buoys satisfied the collocation criteria, only the measurements from the buoy nearest to the corresponding ASAR WM data location were used for the validation and calibration. Locations of the collocated ECMWF buoys and NDBC buoys data are shown in Fig. 2.

### Comparison of ASAR retrievals with buoy wave data

**Fig. 3:**
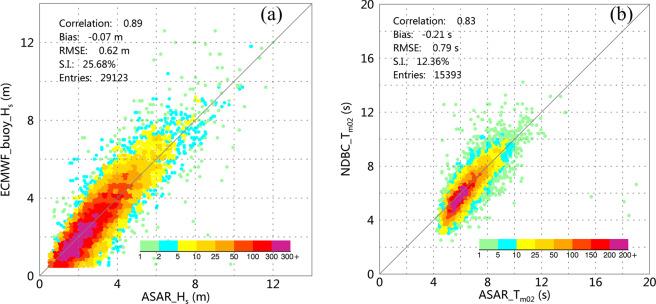
(**a**) Comparison between the ASAR-derived SWH and the ECMWF buoy data. (**b**) Comparison between the ASAR-derived MWP and the NDBC buoy data.

To compare the ASAR-derived SWH (denoted ASAR_*H*_*s*_) with the ECMWF buoy SWH (denoted *ECMWF*_*buoy*_*H*_s_), we limited the SWH to the range from 0.5 m (to avoid the biased retrievals induced by the very low radar backscatter of the sea surface) to 30.0 m. Eventually, 29,123 data pairs were retained for comparison, and the corresponding scatter diagram is shown in Fig. 3(a). With respect to the comparison of MWP, the NDBC buoy MWP (denoted NDBC_*T*_*m*02_) are all larger than 2.0 s but smaller than 20.0 s. Eventually, 15,393 data pairs were used for calibration and validation, and the corresponding scatter diagram is shown in Fig. 3(b). The colors in the two diagrams indicate the density of data pairs.

The following four statistical parameters were used to evaluate the comparisons of the ASAR-derived (referring to both raw and calibrated) sea state parameters with buoy data or RA data, where *X* represents the ASAR-derived sea state parameters and *Y* represents either the buoy data or the RA data. $N$ is the quantity of collocated data pairs. $\bar{X}$ and $\bar{Y}$ represent the mean values of the variables *X* and *Y*, respectively. The correlation coefficient is calculated by the covariance *Cov* (*X*, *Y*) and the variances *D*(*X*) and *D*(*Y*).

5 $$\begin{array}{rll}Correlation & = & \frac{Cov(X,Y)}{\sqrt{D(X)}\sqrt{D(Y)}}\\ Cov(X,Y) & = & \frac{1}{N}\sum \left({X}_{i}-\bar{X}\right)\left({Y}_{i}-\bar{Y}\right)\\ D\left(X\right) & = & \frac{1}{N}\sum {\left({X}_{i}-\bar{X}\right)}^{2},D\left(Y\right)=\frac{1}{N}\sum {\left({Y}_{i}-\bar{Y}\right)}^{2}\\ Bias & = & \bar{Y}-\bar{X}\\ RMSE & = & \sqrt{\frac{\sum {\left({Y}_{i}-{X}_{i}\right)}^{2}}{N}}\\ {S.I.} & = & \frac{1}{Y}\sqrt{\frac{\sum {[\left({Y}_{i}-\bar{Y}\right)-\left(Xi-\bar{X}\right)]}^{2}}{N}}\end{array}$$

ASAR_*H*_*s*_ is slightly higher than ECMWF_buoy_*H*_*s*_ with a bias of 0.07 m. The RMSE is 0.62 m, which is close to the result (0.70 m) achieved in the preliminary validation based on a two-month dataset^17^. The scatter index (S.I.) of 25.68% is relatively high. Furthermore, a comparison of the MWP results suggests that the ASAR retrievals are also slightly higher than the NDBC buoy-measured periods with a bias of 0.21 s. In contrast, the retrieved ASAR_*T*_*m*02_ are closely distributed both sides of the 1:1 diagonal line, and therefore, the comparison yields a low S.I. of 12.36%. With respect to the correlation coefficient, both comparisons suggest that the ASAR retrievals display good agreement with the ECMWF and NDBC buoy measurements, having values of 0.89 and 0.83, respectively.

### Calibration of the ASAR-derived SWH data

Our goal is to calibrate the ASAR-derived sea state parameters using buoy measurements; however, quite a few collocations are outliers, as illustrated in Fig. 3. If these outliers are included in the calibration process, they can introduce uncertainty. Therefore, we used quartiles to exclude some outliers from the calibration process^29^. Quartiles are obtained by dividing the data sorted in ascending order into four equal groups, which can be used to describe the distribution of the data and identify the outliers. The second quartile *Q*_2_ is the median of the data. The first quartile *Q*_1_ and the third quartile *Q*_3_ represent the data between the median and the minimum and maximum, respectively. *IQR* is the interquartile range. According to *Q*_1_, *Q*_2_, *Q*_3_ and the *IQR*, the lower and upper bounds can be calculated. The data exceeding the lower and upper bounds are regarded as outliers.

6 $$\begin{array}{rll}IQR & = & {Q}_{3}-{Q}_{1}\\ lower\,bound & = & {Q}_{1}-1.5IQR\\ Upper\,bound & = & {Q}_{3}+1.5IQR\end{array}$$

By applying these quartiles to exclude some outliers, which is based on statistical analysis of the collocated data, we further employed robust regression to detect the outliers^30,31^ of the collocated data pairs. Robust regression is a linear regression method that is insensitive to outliers. At the start of the regression, all the fitting data have equal weights. By applying least-square minimization, the predicted values and residuals are calculated, where the residuals represent the difference between the predicted values and the observed ones. The data with large residuals are assigned small weights in the subsequent iterations. After a few iterations, the weights of the fitting data are adjusted, and the outliers are verified to have small weights. In this study, the fitting data with weights smaller 0.15 are considered outliers and are excluded from the calibration of the ASAR SWH data.

**Fig. 4:**
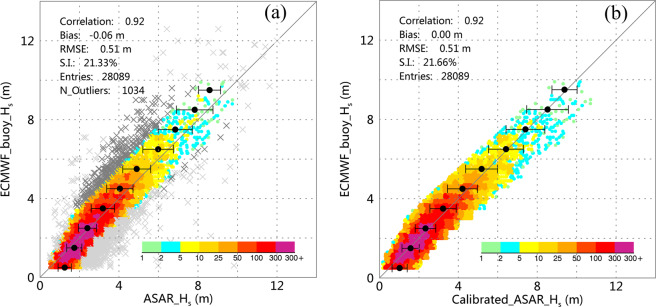
(**a**) Comparison between the ASAR-derived SWH (excluding the detected outliers) and the ECMWF buoy SWH. The light and dark gray cross symbols are the outliers detected by quantile and robust regression methods, respectively. (**b**) Comparison between ASAR-derived SWH and the ECMWF buoy SWH after calibration.

The light and dark gray cross symbols in Fig. 4 represent the outliers detected by the quantile and the robust regression methods, respectively.

Although the quantile and robust regression methods successfully excluded some data pairs as outliers (as indicated by the improved statistical parameters), the comparison shown in Fig. 4(a) suggests that the difference between ASAR_*H*_*s*_ and ECMWF_buoy_*H*_*s*_ is still distinct; specifically, the underestimation of the SWH increases along with sea state varying. In the next step, the buoy measurements were used to calibrate the ASAR retrievals.

The *in situ* measurements are the most appropriate data source of Cal/Val of satellite retrievals. However, these data are not completely unbiased or free of errors^32^. Therefore, we used the reduced major axis (RMA) regression method^31,33^, which treats the variables *x* (ASAR_*H*_*s*_) and *y* (ECMWF_buoy_*H*_*s*_) independently, to calibrate the ASAR retrievals. In the regression, the errors of $x$ and $y$ are both considered by minimizing the triangular area $0.5\times \left(\Delta x\Delta y\right)$ between the data points and the regression line, where Δ*x* and Δ*y* are the distances between the actual and predicted values in the *x* and *y* directions, respectively. By applying RMA regression to the collocated data pairs, the following linear calibration formula for the ASAR SWH data is obtained:

7 $$Calibrated\_ASAR\_{H}_{s}(m)=1.140\times ASAR\_{H}_{s}(m)-0.402$$

Figure 4(b) shows a comparison between the Calibrated_ASAR_*H*_*s*_ and the buoy measurements ECMWF_buoy_*H*_*S*_. The calibration process does improve the bias, which decreases from 0.06 m to zero. However, the other three parameters, including the correlation coefficient, RMSE and S.I., do not improve. Although performing the calibration does not improve the overall statistical parameters, it significantly improves the underestimation of the ASAR-retrieved SWH, as revealed by the error bars overlaid on the scatter diagram, while the underestimation trend originally increases with the wave height. Because the collocated data pairs are unequally distributed among different wave heights and much of the data (62.58%) are associated with a low to moderate sea state (SWH < 2.5 m), the overall statistical parameters do not reflect the effect of calibration on the ASAR-retrieved SWH for different sea states. The following Table 1 lists the variations in the bias and RMSE with the sea state (the Douglas sea scale is used) before and after applying the RMA calibration to the collocated data pairs.

**Table 1:** Variations in the bias (Buoy – ASAR) and RMSE with the sea state before and after the RMA calibration.

Range (m)	Description	No. Collocations	Bias (m)		RMSE (m)	
			Raw	Calibrated	Raw	Calibrated
0.50–1.25	Slight	4548	−0.43	−0.22	0.54	0.44
1.25–2.50	Moderate	13031	−0.16	−0.03	0.40	0.41
2.50–4.00	Rough	7362	0.14	0.13	0.52	0.59
4.00–6.00	Very Rough	2622	0.38	0.18	0.74	0.75
6.00–9.00	High	507	0.46	−0.04	0.89	0.87
9.00–14.00	Very High	19	0.74	−0.06	0.92	0.62

The bias is significantly reduced by the calibration process, particularly for the slight, and higher than rough sea states. This finding indicates that the linear calibration partially reduces the problem of overestimation for slight to moderate sea states and underestimation for rough and high sea states. The RMSE displays slight fluctuations before and after the calibration process, except for the very high sea state (SWH larger than 9.00 m), for which it is reduced by approximately 33% after the calibration.

### Calibration of the ASAR-derived MWP

**Fig. 5:**
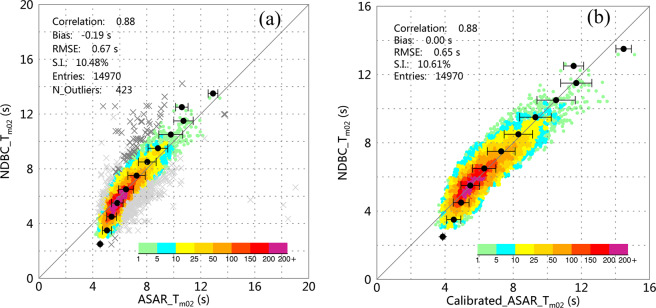
(**a**) Scatter diagram of the comparison between the ASAR-derived MWP and the NDBC buoy MWP. (**b**) the same as (**a**) but for the comparison of the calibrated ASAR-derived MWP. There are few data pairs with large values of MWP that are detected outliers (lower right of (**a**)); therefore, the maximum value of the axes in (**b**) is reduced to 16 s to better visualize the distribution of the error bars. The cross symbols in (**a**) are the detected outliers using the IQR (light gray) and robust regress (dark gray) methods.

Following the same calibration method applied to the SWH, the NDBC buoy data are used to calibrate the ASAR-derived MWP. In total, 15,393 data pairs were collected to calibrate the MWP data considering the collocation criteria mentioned above. After elimination of outliers by the quartile and robust regression methods, 14,970 pairs of data remained. The scatter diagram of the comparison is shown in Fig. 5(a), where the colors represent the density of data pairs and the cross symbols indicate the detected outliers. Using the RMA regression method, a linear calibration of the MWP is derived:

8 $$Calibrated\_ASAR\_{T}_{m02}(s)=1.268\times ASAR\_{T}_{m02}(s)-1.887$$

The calibrated ASAR MWP results are plotted against the NDBC buoy data in Fig. 5(b). Comparing Fig. 5(a) with (b), the calibration improves both the bias and the RMSE, which decrease from −0.19 s to zero and from 0.67 s to 0.65 s, respectively. However, the correlation coefficient and S.I. do not improve. The raw data suggest that the ASAR-derived results overestimate the MWP below 7 s but underestimate it above 8 s. The calibration makes the data pairs almost symmetrically distributed about the 1:1 diagonal line and partially corrects the trend result.

The above-described calibration of the ASAR retrieval is based on collocating buoy data within a 100 km distance. We also tried to reduce the collocation distance to 50 km (consequently, the number of collocation data pairs decreased to 8,046), which yields the following two calibration formulas for the SWH and MWP, respectively, using the same method described above.

9 $$Calibrated\_ASAR\_{H}_{s}(m)=1.132\times ASAR\_{H}_{s}(m)-0.383$$

10 $$Calibrated\_ASAR\_{T}_{m02}(s)=1.256\times ASAR\_{T}_{m02}(s)-1.821$$

The difference in the formulas derived based on a100 km and 50 km collocation distance for calibrating the ASAR-derived SWH and MWP is nearly neglected. For instance, assuming an extreme sea state with the SWH of 20 m, the difference in the calibrated SWH using the two formulas is approximately 0.14 m, which accounts for 0.7% of the SWH. In the provided product, the derived calibration formulas based on a 100 km collocation distance are applied to the ASAR retrieval of SWH and MWP, while the user can easily apply the other set of calibration formulas in (9) and (10) for exploitation.

## Data Records

The ASAR WM data global wave product is stored in NetCDF-3 format and follows the Climate and Forecast Metadata CF-1.7 convention^34^. The naming convention of the ASAR sea state product files is as follows:

Satid_Sensor_Type_StartDate_StartTime_EndDate_EndTime_Cycle_Orbit.NC, where

a.Satid: mission name

b.Sensor: sensor name

c.Type: type of product

d.StartDate: Date of the first record

e.StartTime: Time of the first record

f.EndDate: Date of the last record

g.EndTime: Time of the last record

h.Cycle: cycle number of the satellite

i.Orbit: relative orbit number of the satellite

The records contained in the product correspond to the imagettes of the ASAR WVI Level 1B product. Each record consists of 14 variables, which are listed in the following Table 2.

**Table 2:** List of variables and their descriptions in the ASAR WM sea state NetCDF product.

No.	Variables	Description
1	time	Acquisition time of the ASAR imagettes. Seconds since 2000–01–01 00:00:00 UTC
2	latitude	Latitude of ASAR imagette center
3	longitude	Longitude of ASAR imagette center
4	heading	Flight direction of the satellite (clockwise relative to north)
5	inci_angle	Local incidence angle of ASAR imagette center
6	land_flag	0B for ocean area 1B for land area
7	homogeneity	Homogeneity of ASAR imagettes
8	normalized_variance	Normalized variance of ASAR imagettes
9	rejection_flag	The records flagged 0B are acceptable ASAR imagettes for retrieval 1B for a “bad_record” 2B for “land” 3B for “inhomogeneous ASAR imagettes” 4B for “ASAR imagettes in HH polarization” 5B for “ASAR imagettes with an incidence angle not equal to 23°” 6B for “ASAR imagettes in polar regions, i.e. beyond 70°N or 65°S”
10	qc_flag	0B for a good record 1B for a suspect record 2B for a bad record
11	swh	Retrieved SWH of ASAR imagettes
12	mwp	Retrieved zero-upcrossing wave period of ASAR imagettes
13	swh_cali	Calibrated SWH
14	mwp_cali	Calibrated zero-upcrossing MWP

The “*swh*” and “*mwp*” are the retrieved sea state parameters using the CWAVE_ENV model. By applying the calibration formulas given in Eqs. (7) and (8), the calibrated ASAR-derived SWH and MWP are obtained and stored as the variables “*swh_cali*” and “*mwp_cali*”.

**Fig. 6:**
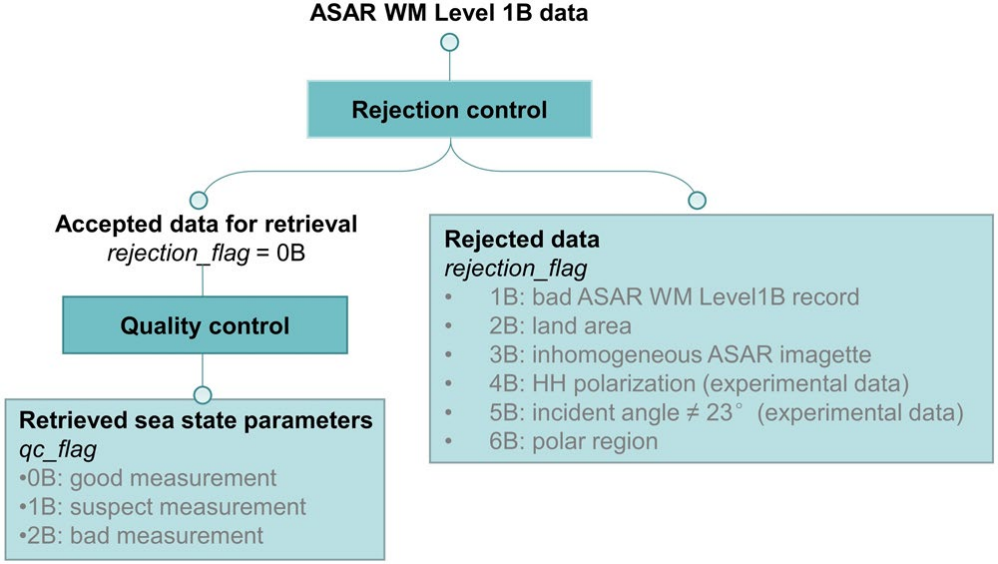
Structure of the “*rejection_flag*” and “*qc_flag*” of the developed global ASAR WM wave products.

The diagram shown in Fig. 6 illustrates the structure of the “*rejection_flag*” and “*qc_flag*” designed in the product.

The “*rejection_Flag*” flags mark the ASAR WM records with values of 0, 1, 2, 3, 4, 5 or 6, which represent an acceptable ASAR WM imagette for retrieval, a bad record (identified in reading the Level 1B data), a record containing land (discrimination is based on “*land_flag*”), an inhomogeneous (homogeneity factor > 1.5) ASAR imagette, an imagette acquired in HH polarization, an imagette with an incidence angle not equal to 23°, and an imagette acquired in the polar regions, respectively. The “*land_flag*” is inherited from the ASAR WM Level 1B data, i.e., each imagette in the Level 1B data has been flagged “land” or not. The “*normalized_variance*”^25^ variable is the normalized variance of the ASAR WM intensity data and is calculated according to Eq. (11).

11 $$\begin{array}{rll}noramalized\_variance & = & \frac{{I}_{var}}{{I}_{mean}\times {I}_{mean}}\\ {I}_{mean} & = & \frac{{\sum }_{i,j=1}^{M,N}{I}_{i,j}}{M\times N}\\ {I}_{var} & = & \frac{{\sum }_{i,j=1}^{M,N}{\left({I}_{i,j}-{I}_{mean}\right)}^{2}}{M\times N}\end{array}$$

where *I*_*var*_ and *I*_*mean*_ represent the variance and mean of the image, respectively, and *M* and *N* refer to the width and height of the image, respectively.

The “*qc_flag*” variable has three values that describe the quality of the retrieved sea state parameters. We considered a few factors during the quality control process, including the reasonable range of retrievals, the normalized variance of the original ASAR intensity image and the “*rejection_flag*”. Based on the factors, the records were assigned different flags.

1. Good record (*qc_flag* = 0B), which satisfies the following criteria:

- 0 m ≤ *swh* (*swh_cali*) < 30 m and 0 s < *mwp* (*mwp_cali*) < 20 s
- $$\bar{{\sigma }_{0}}$$ -NESZ > 3 dB
- rejection_flag = 0B
- homogeneity factor < 1.05

where $\bar{{{\rm{\sigma }}}_{0}}$ is the mean normalized radar cross section of the ASAR imagettes and NESZ is the noise equivalent sigma zero, i.e., the noise floor of the ASAR WM data.

(2) Suspect record (*qc_flag* = 1B), which satisfies the following criterion:
  a.*swh* > 30 m or *mwp* > 20 s
  b.1.05 ≤ homogeneity factor ≤ 1.5
  Among the ASAR collocations with buoy data, there are 1,416 data pairs with homogeneity factors between 1.05 and 1.50. Their comparison with the ECMWF buoy SWH has a bias of −0.26 m, an RMSE of 1.02 m, and a correlation of 0.63. Although these statistical parameters are obviously worse than the comparison achieved using the ASAR WM data with homogeneity factors less than 1.05 (Fig. 3(a)), a large portion of these data still have good consistency with the buoy measurements. If the collocation data pairs with homogeneity factors larger than 1.50 (the amount is 677) are compared with the ECMWF buoy SWH, a correlation of only 0.31 and a large S.I. of 79.20% are found. Therefore, we assign the “*qc_flag*” of the ASAR retrievals with homogeneity factors between 1.05 and 1.50 to “*suspect*”, indicating that these records require further investigation.
(3) Bad record (*qc_flag* = 2B), which satisfies one of the following conditions:

a.*swh* (*swh*_*cali*) < 0 m or *mwp* (*mwp*_*cali*) < 0 s

b.$$\bar{{{\rm{\sigma }}}_{0}}$$ -NESZ ≤ 3 dB

Any record with the variable “*rejection_flag*” not equal to 0B is excluded from further processing, and the “*qc_flag*” is therefore set to “_*Fillvalue*”.

Each ASAR WM Level 1B data with a filename extension of “.N1” that we received from the ESA is processed to a NC record. All the NC records in the same year are compressed to a single file with the file extension “.tar.gz”; therefore, there are together 11 GNU zip files corresponding to the data from the year 2002 to 2012. They have been uploaded to the public repository Sea Scientific open data publication (SEANOE, https://www.seanoe.org) with full free access^35^.

## Technical Validation

### Comparison with RA wave data

The GlobWave project also collected wind and wave data for the Geodetic Satellite (GEOSAT), GEOSAT Follow-on (GFO), ERS-1, ERS-2, TOPEX/POSEIDON, JASON-1, JASON-2 and CryoSAT-2 RA missions, with a time span from 1985 onwards. The JASON-1 mission provided wave data from December 2001 until July 2013, which covers the lifetime of the ASAR instrument. GlobWave reprocessed the original JASON-1 measurements and provided quality control flags and calibrated SWH measurements. In this study, we used calibrated Ku-band SWH measurements of JASON-1 to perform a cross-validation with the calibrated ASAR-derived SWH. A quality flag named “*swh_quality*” provided in the GlobWave RA products is used to filter the JASON-1 SWH data with high quality for validation. This flag has three values, namely, 0, 1, and 2, representing a “*good_measurement*”, “*acceptable_for_some_applications*” and “*bad_measurement*”, respectively. Only the data flagged as “*good_measurement*” are used for validation. The same collocation criteria employed in the collocation of buoy data were utilized between the ASAR data and the JASON-1 data.

**Fig. 7:**
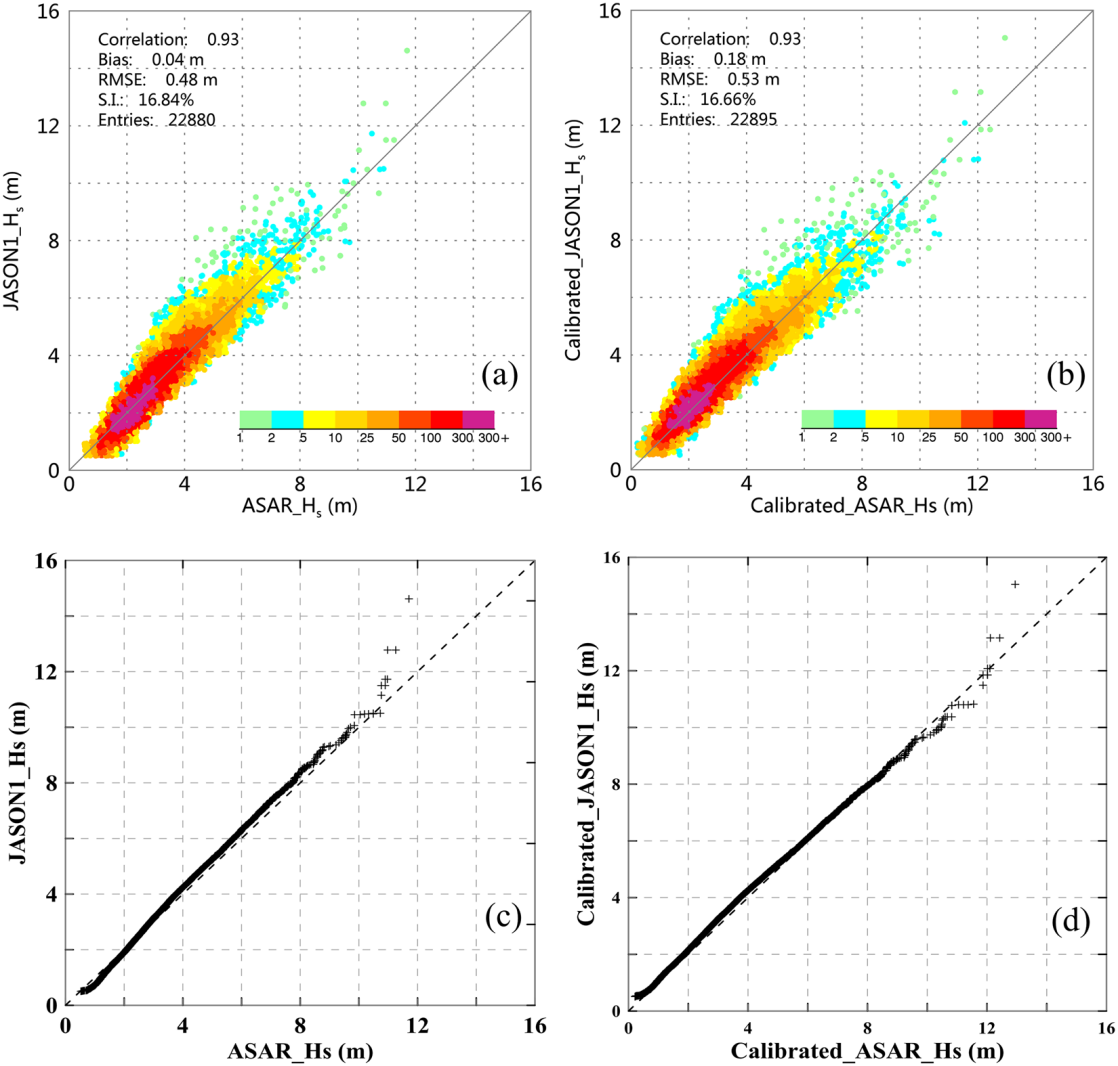
(**a**,**c**) Comparison between the ASAR-derived SWH and the RA SWH along with the corresponding Q-Q plot, respectively. (**b**,**d**) Comparison between the calibrated ASAR-derived SWH and the calibrated RA SWH along with the corresponding Q-Q plot, respectively.

Following the same criteria of collocating the ASAR with buoy, 46,642 data pairs of JASON-1 and ASAR WM data were obtained. However, a large number of SWH measurements from JASON-1 of the GlobWave product in 2012 were abnormal, ranging from −40 m to 40 m and exhibiting a discontinuous spatial distribution. The data in 2012 were discarded from the validation dataset. In addition, we set a valid range of SWH from 0.5 m to 30 m for validation. Finally, 23,192 pairs of JASON-1 and ASAR WM data were obtained. Using the quartile method described above to exclude outliers, 22,880 pairs of data were collected for validation. As there are only few data available for SWH above 10 m, the quartile method of detecting outliers does not function in that range. However, we retain them for comparison. Figure 7(a) shows the comparison between the ASAR-derived SWH and JASON-1 SWH (denoted JASONI_Hs). The robust regression method was not applied to exclude outliers because we consider both datasets to comprise independent measurements. The calibrated ASAR SWH (applying Eq. (7)) is also compared with the JASON-1 calibrated SWH^35^ (Calibrated_JASONI_Hs), as shown in Fig. 7(b).

As shown in Fig. 7(a), the ASAR SWH displays good consistency with the JASON-1 SWH, and the bias and RMSE are 0.04 m and 0.48 m, respectively; additionally, the correlation coefficient and S.I. are 0.93 and 16.84%, respectively. Although the ASAR SWH is generally slightly lower than the JASON-1 SWH, it is higher for a relatively low sea state (SWH < 2.5 m). In Fig. 7(b), the calibrated ASAR SWH also displays good agreement with the calibrated JASON-1 SWH, with bias, RMSE, correlation coefficient and S.I. values of 0.18 m, 0.53 m, 0.93 and 16.64%, respectively. The Q-Q (quantile-quantile) plots shown in Fig. 7(c,d) suggest that the underestimation of ASAR-derived SWH is significantly improved after the calibration process, particularly for SWH above 6 m.

**Fig. 8:**
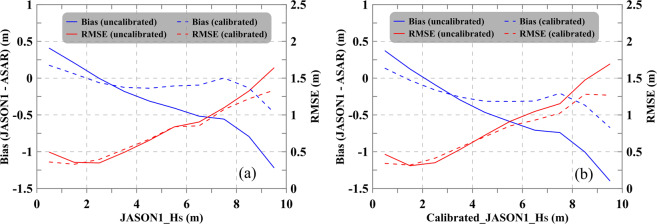
Variations in the bias and RMSE of the ASAR-derived SWH versus the JASON-1 SWH (**a**) and the calibrated JASON-1 SWH (**b**).

A major limitation of these overall comparisons in evaluating the retrieval of sea state parameters is that the data pairs are unevenly distributed among different sea states. As the sea state increases in severity, the number of valid data pairs decreases. Therefore, a stepwise comparison was conducted to assess the performance of the ASAR SWH data quality for different sea states. Figure 8(a) shows the uncalibrated and calibrated ASAR SWH compared with the JASON-1 SWH at a 1-m interval. Figure 8(b) is the same as (a) but compares the ASAR SWH with the calibrated JASON-1 SWH.

Due to the changes in the bias and RMSE illuminated in Fig. 8(a,b) showing similar trends, we use Fig. 8(a) as an example for further description. Figure 8(a) shows the changes in the bias and RMSE of the uncalibrated and calibrated ASAR SWH versus the JASON-1 SWH, where the blue and red lines represent the bias and RMSE, respectively and the solid and dashed lines refer to the comparisons based on the uncalibrated and calibrated ASAR SWH, respectively. The bias of the uncalibrated ASAR SWH increases with the sea state and changes from negative to positive when the SWH is approximately 2 m. The calibration process significantly reduces the bias to less than 0.15–0.2 m from low to high sea states (at approximately 8 m), and importantly, the bias becomes less dependent on the sea state increasing. For a very high sea state (SWH > 9 m), the bias accounts for approximately 10% of the total SWH; additionally, the RMSE of the calibrated ASAR SWH varies from 0.25 m to 1.20 m and is particularly reduced for sea states higher than very rough (above approximately 5 m).

The cross-validation of the ASAR-derived SWH is based on the comparison with the GlobWave JASON-1 data mainly due to that the JASON-1 RA wave data has almost the same temporal coverage as the ASAR WM data in full-life time. Cross-validations with other RA data remain further investigation, e.g., using the recently released comprehensive RA dataset by Ribal and Young^1^, in which there are a few RA missions that also have overlap with the operating period of the ASAR. This can particularly diagnose the accuracy of the ASAR SWH of high sea state, as found in Fig. 7.

Thus far, there is no other high-quality MWP dataset by spaceborne remote sensing available for cross-validation of the ASAR-derived MWP. Further investigation by carefully selecting a reanalysis wave model dataset might be worth trying.

## Supplementary information

Supplementary Information

Supplementary Information 2

## Data Availability

Both the MATLAB code script named read_AGWD.m and the IDL code named read_AGWD.pro for reading the ocean wave parameter products are provided as supplementary material 1 and 2, respectively.
