# Supplementary material for: A global sea state dataset from spaceborne synthetic aperture radar wave mode data
Source: Sci Data. 2020 Aug 7;7:261. doi: 10.1038/s41597-020-00601-3 (PMC7415138; doi:10.1038/s41597-020-00601-3)
Supplement: Supplementary file 1 — Supplementary Information [file 41597_2020_601_MOESM1_ESM.pdf]

```
pro read_AGWD
  ; This producer is used to read the ASAR Wave mode Global sea state Dataset (AGWD)
  ; Author: Bingqing Huang
  ; Affiliation: Aerospace Information Research Institution, Chinese Academy of Sciences
  ; contact: huangbq@aircas.ac.cn

  ; pick up the file to read
  filename = dialog_pickfile()

  ; open file
  fid = ncdf_open(filename, /nowrite)

  ; structure of NC file
  file_structure = NCDF_INQUIRE(fid)

  ; show the global attributes
  print, '                  Global Attributes                '
  print, '---------------------------------------------------'
  print, '---------------------------------------------------'
  for i= 0, file_structure.NGATTS - 1 do begin
    attname = ncdf_attname(fid, i, /global)
    ncdf_attget, fid, attname, attvalue,/global
    print, attname, ':', string(attvalue)
  endfor
  print, '---------------------------------------------------'
  print, '---------------------------------------------------'

  ; read variables
  ; time: second since 2000-01-01 00:00:00 UTC
  time_id = ncdf_varid(fid, 'time')
  ncdf_varget, fid, time_id, time
  ; latitude
  lat_id = ncdf_varid(fid, 'latitude')
  ncdf_varget, fid, lat_id, lat
  ; longitude
  lon_id = ncdf_varid(fid, 'longitude')
  ncdf_varget, fid, lon_id, lon
  ; satellite heading direction in degrees clockwise from North
  heading_id = ncdf_varid(fid, 'heading')
  ncdf_varget, fid, heading_id, heading

  ; incidence angle
  incidence_id = ncdf_varid(fid, 'inci_angle')
  ncdf_varget, fid, incidence_id, inci_angle
  ; land_flag, 0 for ocean 1 for land
  land_id = ncdf_varid(fid, 'land_flag')
  ncdf_varget, fid, land_id, land_flag
  ; homogeneity of imagette
  homog_id = ncdf_varid(fid, 'homogeneity')
```

```
  ncdf_varget, fid, homog_id, homogeneity
  ; Normalized_variance of SAR image
  variance_id = ncdf_varid(fid, 'normalized_variance')
  ncdf_varget, fid, variance_id, normalized_variance
  ; rejection flag,
  ; 0B -- acceptable ASAR WM imagette
  ; 1B -- bad ASAR WM record
  ; 2B -- land
  ; 3B -- inhomogeneous WM imagette (inhomogeneous GT 1.5)
  ; 4B -- H-H polarization (experimental period)
  ; 5B -- incidence angle NE 23 (experimental period)
  ; 6B -- polar region (beyond 65° S or 70° N)
  rejection_id = ncdf_varid(fid, 'rejection_flag')
  ncdf_varget, fid, rejection_id, rejection_flag
  ; qc_flag
  ; 0B -- good
  ; 1B -- suspect
  ; 2B -- bad
  qc_id = ncdf_varid(fid, 'qc_flag')
  ncdf_varget, fid, qc_id, qc_flag
  ; swh
  swh_id = ncdf_varid(fid, 'swh')
  ncdf_varget, fid, swh_id, swh
  ; mwp
  mwp_id = ncdf_varid(fid, 'mwp')
  ncdf_varget, fid, mwp_id, mwp
  ; swh_cali
  swhcali_id = ncdf_varid(fid, 'swh_cali')
  ncdf_varget, fid, swhcali_id, swh_cali
  ; mwp_cali
  mwpcali_id = ncdf_varid(fid, 'mwp_cali')
  ncdf_varget, fid, mwpcali_id, mwp_cali

  ncdf_close, fid

end
```
