# Supplementary material for: A global sea state dataset from spaceborne synthetic aperture radar wave mode data
Source: Sci Data. 2020 Aug 7;7:261. doi: 10.1038/s41597-020-00601-3 (PMC7415138; doi:10.1038/s41597-020-00601-3)
Supplement: Supplementary file 2 — Supplementary Information 2 [file 41597_2020_601_MOESM2_ESM.pdf]

```matlab
% this program is used to read the ASAR Global Wave Dataset
%
% Author: Bingqing Huang
% Affiliation: Aerospace Information Research Institution
%              Chinese Academy of Sciences
% e-mail: huangbq@aircas.ac.cn
% Date of creation: 16th, Jan., 2020

clear all;

%% User input
% please choose the NC file to read
[Filename,File_path]=uigetfile('*.NC','Please choose the NC file to read');
infile=[File_path,Filename];

if ~exist(infile)
    error('the file does not exist!');
end


%% read variables

heading = ncread(infile, 'heading');                    % local satellite heading(clockwise relative to North)

homogeniety = ncread(infile, 'homogeneity');            % homogeneity of ASAR imagette

inci_angle = ncread(infile, 'inci_angle');              % local incidence angle at the sea surface

land_flag = ncread(infile, 'land_flag');                % land flag(0 for ocean, 1 for land)

lat = ncread(infile, 'latitude');                       % latitude of ASAR imagette center, from -90 to 90 degrees
                                                        % positive Latitude is north latitude

lon = ncread(infile, 'longitude');                      % longitude of ASAR imagette center, from -180 to 180 degrees
                                                        % positive Longitude is east Longitude

mwp = ncread(infile, 'mwp');                            % up-crossing zero mean wave period derived from ASAR WM data

mwp_cali = ncread(infile, 'mwp_cali');                  % calibrated up-crossing zero mean wave period

normalized_variance = ncread(infile, ...                % normalized variance of an ASAR imagette
    'normalized_variance');

qc_flag = ncread(infile, 'qc_flag');                    % quality control flag
                                                        % 0 for a good data record
                                                        % 1 for a suspect data record
                                                        % 2 for a bad record

```

```
rejection_flag = ncread(infile, 'rejection_flag');  % rejection flag
                                                    % 0 for acceptable record
                                                    % 1 for a bad record
                                                    % 2 for land
                                                    % 3 for inhomogeneous ASAR imagette
                                                    % 4 for ASAR imagette in HH polarization
                                                    % 5 for ASAR imagette with an incidence
                                                    % angle not equal to 23бу
                                                    % 6 for ASAR imagette in the polar regions,
                                                    % i.e. beyond 70 degree North or 65 degree South

swh = ncread(infile, 'swh');                        % significant wave height derived from ASAR using CWAVE_ENV model

swh_cali = ncread(infile, 'swh_cali');              % calibrated significant wave height

time = ncread(infile, 'time');                      % acquisition time of the ASAR imagettes
                                                    % seconds since 2000-01-01 00:00:00 UTC
%converting the seconds to Date and Time
cal_time = datetime(2000, 1, 1 , 0, 0, 0) + seconds(time);
```
